# Fat-specific Dicer deficiency accelerates aging and mitigates several effects of dietary restriction in mice

**DOI:** 10.18632/aging.100970

**Published:** 2016-05-28

**Authors:** Felipe C. G. Reis, Jéssica L. O. Branquinho, Bruna B. Brandão, Beatriz A. Guerra, Ismael D. Silva, Andrea Frontini, Thomas Thomou, Loris Sartini, Saverio Cinti, C. Ronald Kahn, William T. Festuccia, Alicia J. Kowaltowski, Marcelo A. Mori

**Affiliations:** ^1^ Department of Biophysics, Escola Paulista de Medicina, Universidade Federal de São Paulo, São Paulo, Brazil; ^2^ Department of Gynecology, Escola Paulista de Medicina, Universidade Federal de São Paulo, São Paulo, Brazil; ^3^ Department of Public Health, Experimental and Forensic Medicine, University of Pavia, Pavia, Italy; ^4^ Section on Integrative Physiology and Metabolism, Joslin Diabetes Center, Harvard Medical School, Boston, MA 02215, USA; ^5^ Department of Clinical and Experimental Medicine, Università Politecnica delle Marche, Ancona, Italy; ^6^ Departament of Physiology, Instituto de Ciências Biomédicas, Universidade de São Paulo, São Paulo, Brazil; ^7^ Department of Biochemistry, Instituto de Química, Universidade de São Paulo, São Paulo, Brazil; ^8^ Department of Biochemistry and Tissue Biology, Instituto de Biologia, Universidade Estadual de Campinas, Campinas, Brazil

**Keywords:** dicer, adipose tissue, aging, dietary restriction, insulin resistance

## Abstract

Aging increases the risk of type 2 diabetes, and this can be prevented by dietary restriction (DR). We have previously shown that DR inhibits the downregulation of miRNAs and their processing enzymes - mainly Dicer - that occurs with aging in mouse white adipose tissue (WAT). Here we used fat-specific Dicer knockout mice (AdicerKO) to understand the contributions of adipose tissue Dicer to the metabolic effects of aging and DR. Metabolomic data uncovered a clear distinction between the serum metabolite profiles of Lox control and AdicerKO mice, with a notable elevation of branched-chain amino acids (BCAA) in AdicerKO. These profiles were associated with reduced oxidative metabolism and increased lactate in WAT of AdicerKO mice and were accompanied by structural and functional changes in mitochondria, particularly under DR. AdicerKO mice displayed increased mTORC1 activation in WAT and skeletal muscle, where Dicer expression is not affected. This was accompanied by accelerated age-associated insulin resistance and premature mortality. Moreover, DR-induced insulin sensitivity was abrogated in AdicerKO mice. This was reverted by rapamycin injection, demonstrating that insulin resistance in AdicerKO mice is caused by mTORC1 hyperactivation. Our study evidences a DR-modulated role for WAT Dicer in controlling metabolism and insulin resistance.

## INTRODUCTION

Aging is an important risk factor for chronic diseases such as type 2 diabetes (T2D) [1]. Dietary restriction (DR) increases lifespan and delays the onset of T2D in mammals, including humans [2, 3]. This is thought to be a consequence of increased insulin sensitivity and improved glucose disposal, although the mechanisms underlying these effects of DR have not yet been elucidated in detail. Among the proposed mechanisms, DR has been shown to ameliorate oxidative imbalance [4] and inflammation [5, 6] in a variety of tissues, including the white adipose tissue (WAT), contributing therefore to enhance local and whole body insulin signaling [7-9].

WAT plays a major role in glycemic control and in nutrient homeostasis, serving as the main site for calorie storage during the fed state and as the source of circulating free fatty acids during the fasting state [10]. WAT is also a major endocrine organ [11] and the primary site of branched-chain amino acid (BCAA, *e.g.* valine, leucine, and isoleucine) oxidation [12]. Indeed, impaired BCAA metabolism in adipose tissue and BCAA accumulation in the blood stream have been associated with T2D [13].

Dicer is a type III endoribonuclease that processes pre-miRNAs into mature miRNAs and exerts a variety of other functions related to double-stranded RNA processing and degradation [14]. We have previously reported that DR prevents the age-associated downregulation of Dicer in murine WAT, reversing a global decline in miRNAs that occurs with aging [15]. Dicer expression in adipose tissue is also downregulated in response to obesity and lipodystrophy in mice and humans [16-18], and is affected by aging and DR in *C. elegans* in a manner that resembles the phenomenon observed in mouse adipose tissue [15]. Worms overexpressing Dicer in the intestine - the analog of mammalian adipose tissue - are stress resistant, while whole body Dicer loss-of-function mutations render worms short-lived [15]. Fat-specific Dicer knockout (AdicerKO) mice are insulin resistant and hyperglycemic when subjected to high fat diet [16], suggesting that downregulation of Dicer in adipose tissue contributes to aging and age-associated T2D. Here we tested this hypothesis and asked if DR provides beneficial metabolic outcomes through the upregulation of Dicer in WAT. We found that Dicer is required for proper nutrient utilization by the adipose tissue particularly in catabolic states. Moreover, Dicer loss-of-function in adipocytes directly impacts on the accumulation of circulating metabolites that play a role in controlling whole body insulin action. Consequently, DR is unable to improve insulin sensitivity in AdicerKO mice. Finally, these mice exhibit age-dependent insulin resistance and premature mortality, suggesting a critical role of adipose tissue Dicer in the onset of age-related metabolic diseases.

## RESULTS

### Altered serum metabolite profiles in AdicerKO mice

Twelve-week old AdicerKO and Lox mice were maintained on DR or *ad libitum* (AL) regimens and euthanized when fasting at the end of the protocol. As expected, mice on DR lost weight and visceral adiposity, and this was independent of the genotype (Supplementary Fig. 1A and B). AdicerKO mice had larger brown adipose tissue mass and smaller epididymal mal WAT depots when fed AL, as previously described [16], and these differences persisted under the DR condition (Supplementary Fig. 1B). Surprisingly, DR promoted more subcutaneous inguinal WAT (henceforth referred to simply as WAT) loss in AdicerKO than in the Lox mice (Supplementary Fig. 1B).

**Figure 1:**
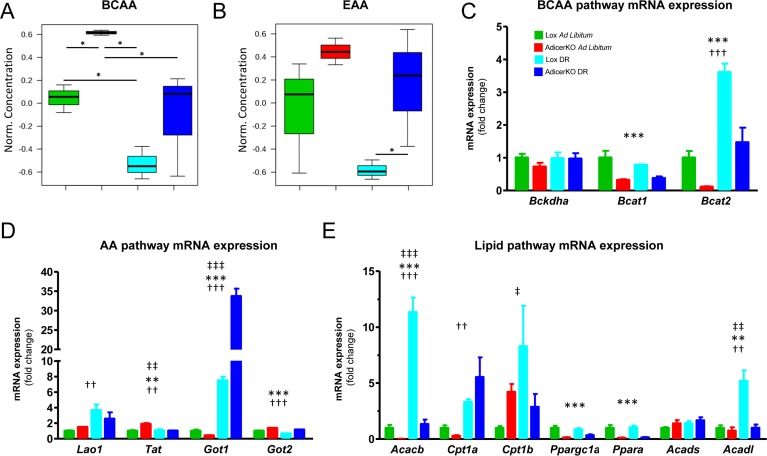
Metabolic changes in fat-specific Dicer knockout mice (AdicerKO) Twelve-week old mice were subjected to *ad libitum* (AL) or dietary restriction (DR) regimens for three months. Mice were euthanized at the end of the protocol after overnight fasting and serum (**A**) branched-chain amino acid (BCAA) or (**B**) essential amino acid (EAA) levels were assessed (N=3 per condition). Values of individual amino acids were summed, normalized by the average of the Lox AL group, Log2 transformed and Pareto scaled. Data are mean ± SE. * P < 0.05. (**C**-**E**) Gene expression in inguinal white adipose tissue (N=5-7 per condition). Mean ± SEM. ** P < 0.01, *** P < 0.001 for genotype effect; †† P < 0.01, ††† P < 0.001 for diet effect; ‡‡ P < 0.01, ‡‡‡ P < 0.001 for diet-genotype interaction.

To test if the absence of Dicer in adipocytes could lead to systemic metabolic changes in AL or DR mice, we performed serum metabolomics. Partial least squares discriminant analysis (PLS-DA) (Supplementary Fig. 2A) and hierarchical clustering analysis (Supplementary Fig. 2B) revealed a distinct pattern between the groups, in particular between DR and AL, but also between AdicerKO and Lox mice. Pathway analysis demonstrated that metabolites related to fatty acid oxidation, BCAA degradation and biosynthesis, pantothenate and CoA biosynthesis, aromatic amino acid biosynthesis, and glycerophospholipid metabolism were the most overrepresented among the differentially expressed serum constituents when comparing all conditions (Supplementary Table 1). Dicer knockout in adipocytes did not completely abrogate the effects of DR on the levels of specific serum metabolites; however it did increase the circulating levels of BCAA and other essential amino acids both under AL (Supplementary Table 2) and DR conditions (Fig. 1A and B). Short-chain acylcarnitines (SCAC) (Supplementary Fig. 2C and D) and glycerol-phospholipids (Supplementary Fig. 2B) were also higher in the serum of AdicerKO mice under these conditions.

### Metabolic rewiring in the adipose tissue of AdicerKO mice

The changes in circulating BCAA and SCAC levels in AdicerKO mice prompted us to investigate potential differences in the expression of genes related to amino acid and fatty acid metabolism in WAT - a major site for BCAA and fatty acid oxidation [12, 19]. Genes encoding branched-chain aminotransferases (*Bcat1* - cytoplasmic and *Bcat2* -mitochondrial) – the first step in the BCAA degradation pathway - were lower by 52 to 89% in AdicerKO WAT, especially the mitochondrial isoform *Bcat2*, which was also induced by DR in both genotypes (Fig. 1C). Branched-chain alpha-ketoacid dehydrogenase mRNA (*Bckdha*) was not changed (Fig. 1C). We also measured the mRNA expression of enzymes involved in the catabolism of other amino acids, *e.g*. L-amino acid oxidase 1 (*Lao1*), tyrosine aminotransferase (*Tat*), and glutamic-oxaloacetic transaminases (*Got1* – cytoplasmic and *Got2* – mitochondrial) (Fig. 1D). We observed no changes except for *Got1*, which was dramatically increased by 7.5- and 86.3-fold with DR in Lox and AdicerKO mice, respectively. Acetyl-CoA carboxylase beta mRNA (*Acacb*) expression was markedly reduced in the WAT of AdicerKO mice under both AL and DR conditions, despite minor or no consistent changes in mRNA expression of carnitine palmitoyltransferase Ia (*Cpt1a*) and Ib (*Cpt1β*) or short chain fatty acid acyl-coenzyme A dehydrogenase (*Acads*) (Fig. 1E). The mRNA encoding for long chain fatty acid acyl-coenzyme A dehydrogenase (*Acadl*) was lower in the WAT of AdicerKO mice only during DR (Fig. 1E). Taken together with elevated serum SCAC levels in AdicerKO mice (Supplementary Fig. 2C and D) with no changes in palmitoylcarnitine (Supplementary Fig. 2E) or carnitine (Supplementary Fig. 2F), these results suggest reduced BCAA catabolism and altered fatty acid oxidation. In agreement with this hypothesis, two of the major transcription factors involved in the regulation of genes of the mitochondrial β-oxidation, *i.e*. PGC-1α and PPAR-α, had their mRNAs dramatically decreased in WAT of AdicerKO mice in both AL and DR regimens (*Ppargc1a* and *Ppara* - Fig. 1E).

**Figure 2:**
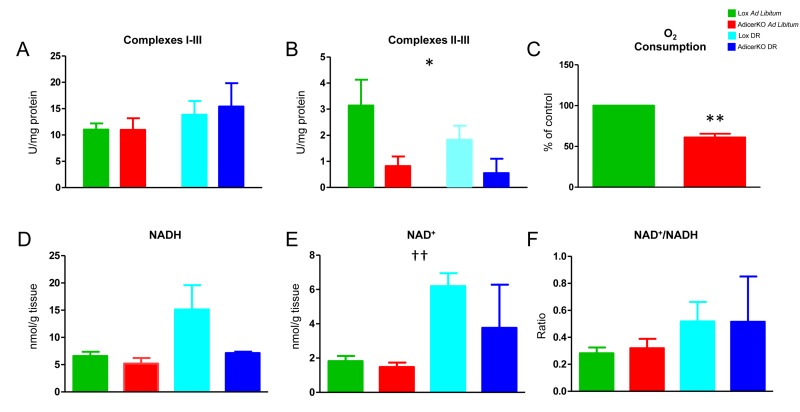
Respiration in white adipose tissue of AdicerKO mice Twelve-week old mice were subjected to *ad libitum* (AL) or dietary restriction (DR) regimens for one month. Inguinal white adipose tissue (WAT) was isolated and cytochrome c reduction was measured when NADH (**A**) or succinate (**B**) was used as substrate to assess ETC complex I-III or II-III activity, respectively (N=3-5 per condition). (**C**) Adipocytes were isolated and succinate-induced oxygen consumption was measured (N=4 pools of at least 2 animals per condition). (**D**) NADH, (**E**) NAD^+^ and (**F**) NAD^+^/NADH ratio were determined in whole WAT (representative of two independent experiments with N=2-6 per condition in each experiment). Values are mean ± SEM. * P < 0.05, ** P < 0.01 for genotype effect; †† P < 0.01 for diet effect.

Surprisingly, isolated WAT or skeletal muscle of AdicerKO mice were able to efficiently oxidize valine (Supplementary Fig. 3A-H) or palmitate (Supplementary Fig. 3I-L) into CO_2_, or direct their carbons to ward lipid synthesis (Supplementary Fig. 3M-R), independently of the diet, when these substrates were offered in excess as an exogenous energy source. These data suggest that oxidative capacity of AdicerKO WAT and muscle is not compromised. Indeed, the capacity to reduce cytochrome c was preserved in the WAT of AdicerKO mice when NADH was offered as a substrate to promote electron transport starting at complex I (Fig. 2A). However, electron transport was less efficient in AdicerKO WAT when succinate was used to feed complex II directly (Fig. 2B). These results indicate that electron transport function is impaired at the level of complex II in AdicerKO WAT. Consistently, AdicerKO adipocytes displayed lower respiratory rates in the presence of succinate (Fig. 2C), indicating that fat cells in which Dicer was knocked out engage less in oxidative metabolism.

**Figure 3:**
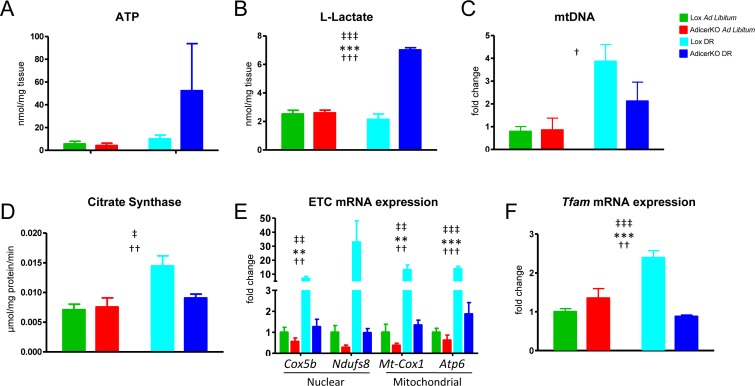
Mitochondrial function and biogenesis in adipose tissue of AdicerKO mice Twelve-week old mice were subjected to *ad libitum* (AL) or dietary restriction (DR) regimens for one (**A,B,D**) or three (**C,E,F**) months. WAT was isolated and (**A**) ATP and (**B**) L-lactate levels were measured in protein-free extracts (N=3-4 per condition); (**C**) mitochondrial DNA (mtDNA) (normalized by nuclear DNA) was assessed by qPCR (N=3 per condition); (**D**) Citrate Synthase activity was determined in protein extracts (N=4-5 per condition); and (E-F) gene expression was quantitated by RT-qPCR (N=4 per condition). Mean ± SEM. ** P < 0.01, *** P < 0.001 for genotype effect; † P < 0.05, †† P < 0.01, ††† P < 0.001 for diet effect; ‡ P < 0.05, ‡‡ P < 0.01, ‡‡‡ P < 0.001 for diet-genotype interaction.

Decreased respiration in the WAT of AdicerKO mice could be caused by low substrate availability. The levels of NADH – the major substrate for the electron transport chain (ETC) – did not differ in WAT of Lox vs. AdicerKO mice, but trended upwards more dramatically in Lox compared to AdicerKO mice with DR, although these differences did not reach statistical significance (Fig. 2D). NAD^+^ levels were similarly increased with DR independently of the genotype (Fig. 2E) and no differences were observed in the NAD^+^/NADH ratios (Fig. 2F). ATP levels were similar between the groups (Fig. 3A), while lactate levels were 3.3-fold higher in AdicerKO mice subjected to DR when compared to the Lox controls on the same regimen (Fig. 3B), indicating a counterintuitive shift towards anaerobic metabolism in AdicerKO mice during a state of negative energy balance.

### Reduced mitochondrial biogenesis in AdicerKO WAT under DR

We next hypothesized that reduced oxidative metabolism could be linked to a reduction in mitochondrial mass and/or number in the WAT of AdicerKO mice. Mitochondrial DNA (mtDNA), a marker of mitochondrial mass, was reduced in the WAT of AdicerKO mice fed AL when compared to the Lox controls, and increased by a similar level in both genotypes upon DR (Fig. 3C). Citrate synthase level - a second mitochondrial mass marker - was not different when comparing Lox and AdicerKO mice under the AL regimen; however it increased significantly in response to DR in Lox but not in AdicerKO WAT (Fig. 3D). Similar patterns were observed when we measured the expression of nuclear- and mitochondria-encoded genes of the ETC (Fig. 3E), as well as the mitochondrial transcription factor *Tfam* (Fig. 3F).

Consistent with a causal role of Dicer in promoting WAT mitochondrial biogenesis in response to DR, and reinforcing the importance of this protein in the early events of the dietary intervention, both Dicer protein levels and the mitochondrial complex I marker *Ndufs8* were upregulated by as early as 3.5-days of DR (Supplementary Fig. 4 A-C). Dicer expression was further increased with time and reached maximum upregulation within 10.5-days of DR (Supplementary Fig. 4B). In addition, to test if it was the ablation of Dicer in adipocytes or the consequent age-dependent lipodystrophic phenotype of AdicerKO mice that blocked the effect of DR over mitochondrial biogenesis in WAT, we studied the effect of the regimen in young mice, before the onset of lipodystrophy [16]. Consistent with a direct role of Dicer in mitochondrial biogenesis, DR induced the expression of ETC genes in WAT of 8-week old Lox mice, but not in aged matched AdicerKO mice (Supplementary Fig. 4D). These data demonstrate that DR promotes a robust induction of mitochondrial biogenesis in WAT that is blocked by adipocyte-specific Dicer loss-of-function.

**Figure 4:**
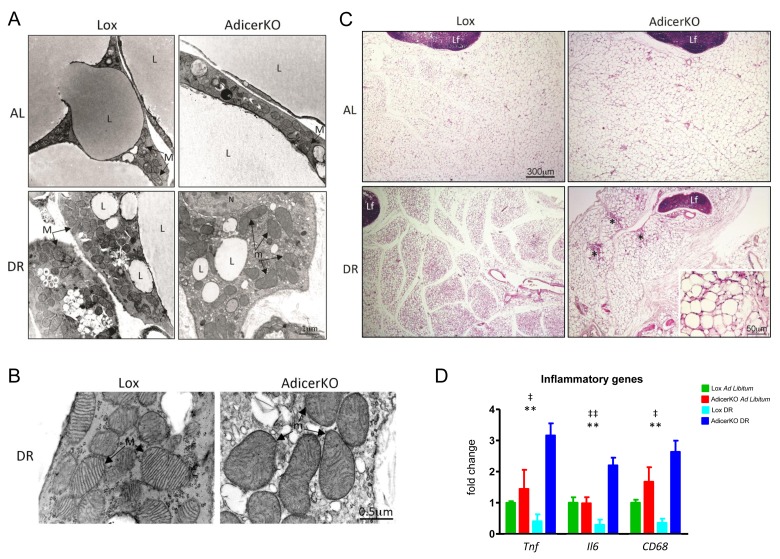
Morphology of white adipose tissue of AdicerKO mice Twelve-week old mice were subjected to *ad libitum* (AL) or dietary restriction (DR) regimens for one (**A**-**C**) or three (**D**) months. Inguinal white adipose tissue (WAT) was isolated and analyzed at the (A,B) ultrastructure level (electron microscopy) or at the (C) histological level (H&E) (N=4-5 animals per condition). L, lipid droplet. M, healthy mitochondrium. m, damaged hypertrophic mitochondrium. Lf, lymph node. N, nucleus. *, fibrosis. (D) Inflammation markers were assessed by RT-qPCR (N=3-4 per condition). Mean ± SEM. ** P < 0.01 for genotype effect; ‡ P < 0.05, ‡‡ P < 0.01 for diet-genotype interaction.

In agreement with the molecular data, electron microscopies of the WAT revealed less abundant mitochondria in the WAT of AL-fed AdicerKO mice (Fig. 4A). On the other hand, while DR induced accumulation of healthy-looking mitochondria in the adipocytes of Lox mice, it resulted in fewer mitochondria with highly irregular shapes and aberrant cristae in AdicerKO mice, hallmarks of a dysfunctional organelle (Fig. 4A and B).

### Structural changes in the WAT of AdicerKO mice upon DR

To assess if the differences in substrate utilization and mitochondrial mass were associated with morphological changes in the WAT of AdicerKO mice, we performed histological analyses. Adipocytes of AdicerKO mice tended to be larger and more unilocular than those of Lox mice on the AL diet (Fig. 4C and Supplementary 5A and B), but these differences did not reach statistical significance. Under DR, adipocytes of Lox became multilocular, while the tissue of AdicerKO appeared fibrotic and filled with cell infiltrate in some areas (Fig. 4C). Consistently, markers of inflammation were increased in the WAT of AdicerKO mice upon DR, in contrast with the effect of DR on Lox mice, which decreased inflammation markers (Fig. 4D).

Surprisingly, protein carbonylation (Supplementary Fig. 5C) and glutathione levels (Supplementary Fig. 5D) - markers of oxidative damage - were not changed or minimally affected among the groups.

### Insulin resistance in AdicerKO mice

**Figure 5:**
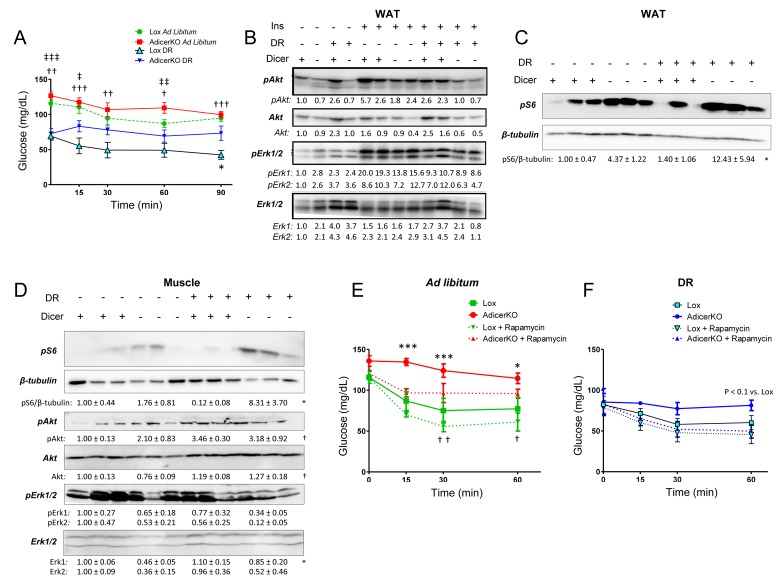
Insulin resistance and mTORC1 hyperactivation in AdicerKO mice Twelve-week old mice were subjected to *ad libitum* (AL) or dietary restriction (DR) regimens for one (**A,B**) or three (**C**-**F**) months. (**A**) Insulin tolerance test (N=5-7 per condition). * P < 0.05 Lox DR vs. AdicerKO DR; † P < 0.05, †† P < 0.01, ††† P < 0.001 Lox AL vs. Lox DR; ‡ P < 0.05, ‡‡ P < 0.01, ‡‡‡ P < 0.001 AdicerKO AL vs. AdicerKO DR. (**B**-**D**) Western blots of tissue extracts when (**B**) insulin was injected in the inferior vena cava and WAT was collected 5 minutes after, or (**C,D**) at basal, random fed state. WAT, inguinal white adipose tissue. Muscle, gastrocnemius. +, the presence of the protein or intervention. -, the absence of the protein or intervention. pS6, phospho-S6. pAkt, phospho-Akt. pErk1/2, phospho-Erk1/2. Numbers are quantitation of blots (fold expression in comparison to control group) ± SEM. * P < 0.05 for genotype effect; † P < 0.05 for diet effect. (**E,F**) Mice subjected to AL (**E**) or DR (**F**) diets were treated with Rapamycin for 2h prior to an insulin tolerance test (N=3-5 per condition). Mean ± SEM. * P < 0.05, *** P < 0.001 Lox vs. AdicerKO; † P < 0.05, †† P < 0.01 Lox + Rapamycin vs. AdicerKO + Rapamycin.

Mitochondrial dysfunction in the adipose tissue and increased inflammation are often associated with insulin resistance [20, 21]. Indeed, AdicerKO mice are insulin resistant, in particular at the level of the adipose tissue [[16] and Fig. 5]. Strikingly, AdicerKO mice remained insulin resistant even under the DR regimen (Fig. 5A), despite showing improved glucose tolerance (Supplementary Fig. 6A) when compared to mice on the AL condition. Whole body insulin resistance in Adicer KO mice was associated with reduced insulin signaling in WAT as measured by Akt and Erk1/2 phosphorylation (Fig. 5B). Interestingly, deletion of Dicer in adipocytes led to downregulation of total Akt levels and selective inhibition of insulin-induced p42 (Erk2) phosphorylation (Fig. 5B). Akt is phosphorylated at Ser473 by the insulin-dependent, rapamycin-insensitive mTORC2 complex [22]. On the other hand, mTORC2 is inhibited by chronic stimulation of the mTORC1 complex, which is in turn activated by BCAA [23]. We thus estimated basal mTORC1 activity by assessing the phosphorylation of the ribosomal protein S6. S6 phosphorylation was markedly increased in WAT of AdicerKO mice, both under the AL and DR regimens (Fig. 5C), indicating hyperactivation of mTORC1. Since we found increased levels of circulating BCAA in AdicerKO mice, we asked whether other non-adipose tissues also showed elevated mTORC1 activity. Indeed, S6 phosphorylation was increased in skeletal muscle of AdicerKO mice under both regimens, despite no changes in Akt or Erk phosphorylation (Fig. 5D). On the other hand, phosphorylation of Erk was reduced in liver of AdicerKO mice with no changes in Akt (Supplementary Fig. 6B).

If hyperactivation of mTORC1 in insulin-sensitive tissues was the cause of insulin resistance in AdicerKO mice, one would expect this phenotype to be reversed by selective inhibition of mTORC1, as previously shown [24]. In agreement with this notion, acute rapamycin injection not only markedly improved insulin sensitivity in AdicerKO mice on the AL diet, but also completely reversed insulin resistance of these mice under the DR regimen (Fig. 5E and F). These results demonstrate that increased mTORC1 activation - possibly due to elevated levels of circulating BCAA - leads to whole body insulin resistance in AdicerKO mice.

### AdicerKO mice exhibit age-associated insulin resistance and increased premature mortality risk

**Figure 6:**
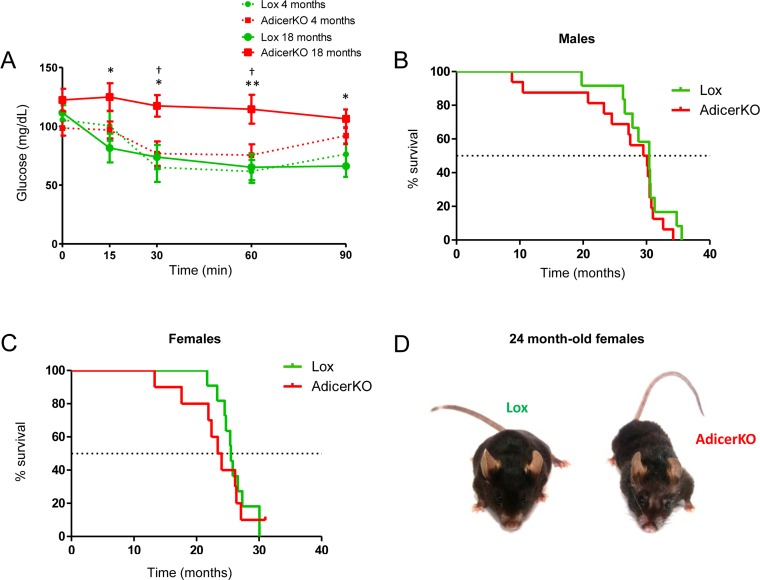
Increased risk of mortality and premature age-related complications in AdicerKO mice (**A**) Insulin tolerance test in young (4-month old) or old (18-month old) male AdicerKO and Lox mice (N=6-7 per condition). Mean ± SEM. * P < 0.05, ** P < 0.01 Lox 18 months vs. AdicerKO 18 months; † P < 0.05 AdicerKO 18 months vs. AdicerKO 4 months. (**B**,**C**) Kaplan-Meier curves of (**B**) male (N=12-16 per genotype) and (**C**) female (N=10-11 per genotype) Lox and AdicerKO mice. (**D**) Representative pictures of 24-month old females. Mice were fed *ad libitum* during these experiments.

Next, to test whether insulin resistance in AdicerKO mice was associated with aging, we performed insulin tolerance tests in young (4 month-old) and old (18-month-old) mice that had AL access to chow during their entire lifespan. Insulin resistance was minimal in young AdicerKO mice, while in old animals it was markedly different (Fig. 6A). We also determined the life expectancy of these mice. Median survival was not different between Lox and AdicerKO mice [Males: Lox vs. AdicerKO = 30.4 vs. 29.8 months, P = 0.3601 (Fig. 6B); Females: Lox vs. AdicerKO = 25.5 vs. 23.7 months, P = 0.5579 (Fig. 6C)], but Gompertz modeling [25] revealed that the initial mortality rate (vulnerability) was significantly higher in AdicerKO mice with no difference in age-dependent mortality (aging rate). Furthermore, female AdicerKO mice exhibited more frequent signs of senescence (such as hair loss or graying) at their median lifespan (24 months of age) than did Lox mice (Fig. 6D). Hence, AdicerKO mice are more prone to early death and trend toward premature aging, but the animals that survive to the last quartile of their lifespan seem to be as sensitive to age-dependent mortality as Lox controls.

## DISCUSSION

**Figure 7:**
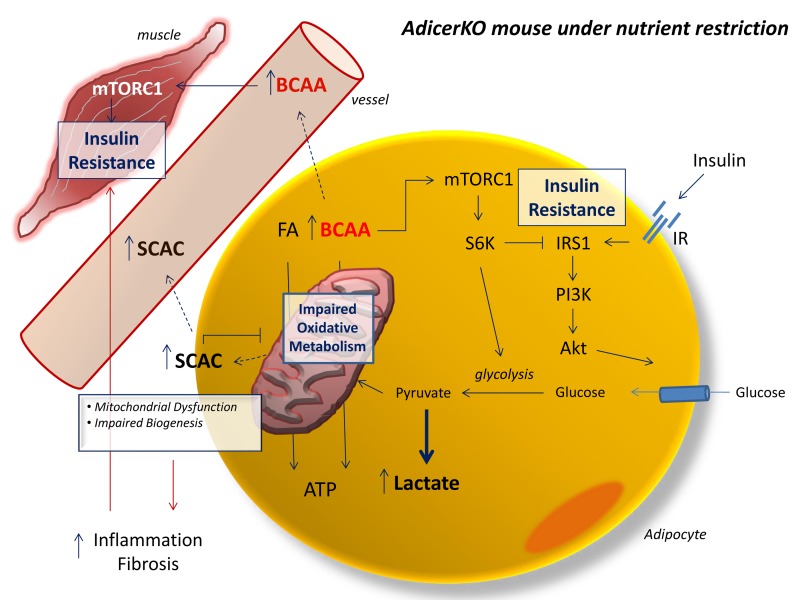
Metabolic dysfunction in AdicerKO mice under nutrient restriction Schematic model of the metabolic consequences of fat-specific Dicer knockout in mice under nutrient restriction. FA, fatty acid. BCAA, branched-chain amino acid. SCAC, short-chain acylcarnitines. IR, insulin receptor. Solid arrows, activation. Dashed arrows, transport. T-bars, inhibition.

The regulation of Dicer expression in the adipose tissue and its consequent impact on global miRNA levels have been proposed to represent an evolutionarily conserved feature of aging and a mechanism through which DR delays the aging process [15, 18]. Here we used mice lacking Dicer in adipocytes (AdicerKO) to directly test these hypotheses. Metabolomic analyses reveal marked changes in the levels of BCAA and SCAC in the blood stream of fasted, middle-aged AdicerKO in comparison to Lox mice, both under the DR and AL regimens. These changes are in agreement with the low oxidative profile of AdicerKO adipocytes. Under short-term DR, the adipose tissue of Lox mice activates a program of mitochondrial biogenesis and oxidative metabolism, whereas the fat tissue of AdicerKO mice shifts its metabolism towards anaerobic glycolysis while abrogating mitochondrial biogenesis. In turn, this leads to tissue dysfunction and inflammation, which contributes to insulin resistance [18, 20, 21, 26] (Fig. 7). Insulin resistance in AdicerKO mice is partially or entirely explained by hyperactivation of mTORC1, depending on the dietary regimen. mTORC1 is activated by BCAA [23], which are elevated in the serum of AdicerKO mice. When activated, mTORC1 leads to phosphorylation of S6 kinase, which in turn phosphorylates IRS1 in a serine residue, therefore inhibiting it and impairing insulin signaling [27, 28]. S6 kinase knockout mice are long-lived and display characteristics that resemble the effects of DR, such as protection from age-dependent insulin resistance [29]. Conversely, high levels of circulating BCAA in obesity and chronic mTORC1 activation have been previously linked to T2D and aging in humans and animal models [13, 30-34]. Another facet of BCAA-induced insulin resistance is related to increased BCAA catabolism in the muscle, which leads to incomplete fatty acid oxidation, gives rise to SCAC that allosterically inhibit citrate synthase, results in mitochondrial stress and impairs insulin action [13]. Hyperactivation of mTORC1 also shifts metabolism towards glycolysis and *de novo* lipid biosynthesis [35], which in sum are phenotypes observed in the AdicerKO mouse under DR. Adipose tissue is a major site for BCAA oxidation and this process is impaired during T2D [12, 13, 19, 30]. Dicer expression is decreased in the adipose tissue of mouse models and humans with increased risk for T2D, such as during obesity, aging and lipodystrophy [15, 16, 18], while increased upon DR [15]. Based on this scenario, we conclude that higher levels of Dicer in adipose tissue are necessary to sustain a proper response to fluctuations in energy demands by conferring adequate substrate utilization and promoting oxidative metabolism. When nutrients are particularly limiting, reduced levels of Dicer negatively impact on adipose tissue oxidative metabolism, raising the blood levels of BCAA and SCAC, contributing to mTORC1 activation and resulting in whole body insulin resistance (Fig. 7). Consistent with this notion, AdicerKO mice do not benefit from some of the classic metabolic outcomes of DR, such as increased insulin sensitivity and decreased inflammation, and have accelerated age-dependent insulin resistance and premature mortality.

Dicer knockout in adipocytes leads to partial lipodystrophy in middle-aged mice as manifested by decreased intra-abdominal fat accumulation and hypertrophy and “whitening” of the interscapular brown fat depot [16]. Since the subcutaneous inguinal fat pad is not grossly affected in size or morphology in chow diet fed, young adult AdicerKO mice [[16] and this study], and given that the impact of aging and DR on Dicer expression is more clearly manifested in this tissue [15], we decided to focus our efforts on this depot. By doing so we wanted to minimize developmental changes in adipose tissue composition and essentially look for changes that occurred in response to DR or aging. Indeed, some of the observations made in 3-month old AdicerKO mice subjected to DR were confirmed in younger mice, prior to the onset of lipodystrophy. These experiments corroborate the notion that Dicer expression in adipocytes is required for a proper response to DR, and not that lipodystrophy mitigates the effect of the diet. In agreement, short term (3.5 days), moderate (10%) DR is able to upregulate Dicer in adipose tissue of wild type mice while also upregulating genes of the mitochondrial ETC, prior to changes in body weight.

The differences in the expression of mitochondrial and β-oxidation-related genes in the adipose tissue of AdicerKO versus Lox mice are particularly evident under DR. Likewise, lactate levels are increased in the fat tissue of AdicerKO mice only in response to this dietary regimen, pointing towards a shift to anaerobic glycolysis during a state of chronic catabolism. Intriguingly, there is a concomitant increase in triglyceride synthesis from valine in the adipose tissue of AdicerKO mice on DR, which suggests diversion of carbons to lipid anabolism. Increases in anaerobic glycolysis and in the flux through anabolic pathways under nutrient limiting states also occur in many cancer cells and are often referred to as the Warburg effect [36]. Interestingly, as in Dicer knockout adipocytes, many of these cancer cells have decreased Dicer levels [37] and reduced oxidative activity [36]. Thus, our data suggest that Dicer knockout adipocytes exhibit a Warburg-like behavior when put on nutrient restriction, somewhat resembling the behavior of cancer cells. This could be linked to the activation of senescence and inflammation pathways that are hallmarks in the adipose tissue of AdicerKO mice and in Dicer knockout preadipocytes (this study and [15]).

The question that remains is why Dicer knockout adipocytes actively divert to non-oxidative metabolism when subjected to nutrient restriction despite having adequate mitochondrial capacity and plenty of substrates. One possible explanation is that under DR, Dicer upregulation in wild type adipocytes leads to the increase of miRNAs that suppress glycolytic genes to favor oxidative metabolism, a phenomenon that would not occur in AdicerKO mice. Dicer levels may be rate-limiting for these miRNAs or their effects may become more evident in a situation when catabolic flux is promoted. Indeed, when lactate or mitochondrial gene expression is assessed in various cell culture models of Dicer knockout preadipocytes, no differences are observed when comparing these cells to the wild type (unpublished data). These cells are cultured and differentiated into adipocytes in an atmosphere of 20% oxygen and in high glucose, high growth factor medium, which is distant from the pseudo-hypoxic, highly oxidative and growth limiting conditions of DR *in vivo*. Moreover, the cells are not exposed to the changes in circulating factors that occur during DR or in response to fat-specific Dicer knockout. Hence, more studies are required to determine the exact mechanism through which Dicer controls adipocyte metabolism during catabolic states.

We and others have shown that Dicer is pivotal in stress responses, metabolic diseases and aging [15, 38, 39]. miRNAs are involved in specific aspects of these mechanisms [39-43], but they are differently expressed, play different roles in distinct cell types and conditions and are unlikely to individually explain the plethora of phenotypes that are characteristic of aging or T2D [44]. Furthermore, while miRNAs are likely to participate as downstream molecular players involved in the phenotypes of AdicerKO mice, Dicer also binds to and directly controls the levels of a wide range of RNAs in the cell, including other non-coding RNAs such as tRNAs or even mitochondrial-encoded mRNAs [14]. Therefore, we propose a scenario where during metabolic stress, Dicer becomes rate-limiting for the biosynthesis of certain RNAs while promoting the degradation of others. These downstream RNAs may vary depending on the cell type, condition or degree of cell differentiation. Dicer upregulation will then favor these processes to promote context-specific adaptation to stress and increase cell robustness [45]. When this occurs in adipocytes, context-specific adaptation to metabolic stress leads to proper substrate utilization and results in efficient nutrient mobilization to metabolic tissues that depend largely on the adipose tissue in situations of calorie deprivation, such as the skeletal muscle. This confers more efficient usage of the scarcely available energy and also contributes to organismal metabolic plasticity, which in turn protects from premature metabolic diseases and eventually death. It also prevents the accumulation of circulating metabolites (such as BCAA) that act as signaling molecules to activate pathways that are positively involved in growth and energy storage while negatively involved in longevity (such as the mTOR pathway) (Fig. 7).

In conclusion, we now provide direct evidence that adipose tissue Dicer is necessary for proper nutrient utilization by adipocytes and essential for the beneficial effects of DR, such as improved whole-body insulin sensitivity. We also demonstrate that adipocyte-specific Dicer knockout is sufficient to accelerate the appearance of insulin resistance in mice, while leading to premature mortality. Our study and the evolutionary conserved association between Dicer regulation, metabolic diseases and aging set the stage for new interventions to prolong healthspan in humans.

## MATERIALS AND METHODS

### Animals

Fat-specific Dicer knockout mice (AdicerKO) and their littermate controls (Lox) were obtained from the Centro de Desenvolvimento de Modelos Experimentais para Medicina e Biologia (CEDEME) of the Universidade Federal de São Paulo. Males were used in all cases unless stated otherwise. Mice were maintained on a 12-hr light-dark cycle with *ad libitum* access to tap water and chow diet. Dietary restriction was performed according to the protocol of the National Institute on Aging [15, 46]. Food intake and body weight was assessed weekly. Mice were euthanized at the indicated time-points during random feeding unless indicated otherwise. After euthanasia, tissues were collected, weighed and immediately used or frozen in dry ice and stored at −80°C. Protocols for animal use were approved by the IACUC of the Universidade Federal de São Paulo (CEP-0218/11, CEP-0237/12 and CEUA4603261015) and were in accordance with NIH guidelines.

### Metabolomics

Serum metabolites were measured by mass spectrometry (BIOCRATES Life Sciences). The results were analyzed using MetaboAnalyst (http://www.metaboanalyst.ca) [47]. Values were normalized by the pooled average value from the Lox AL group, Log2 transformed, and Pareto scaled (mean-centered and divided by the square root of standard deviation of each variable).

### Western blotting and qPCR

These methods were performed as described previously [15, 16]. For mitochondrial DNA quantitation, total DNA was extracted using DNeasy Blood & Tissue kit (Qiagen) and subjected to qPCR using primers targeting mitochondrial or nuclear DNA. Antibodies were: Dicer (Ab13502) from Abcam and phosphor-S6 (5364), Akt (9272), phosphor-Akt (9271), Erk (9102), phospho-Erk (9101), and β-tubulin (2146) from Cell Signaling. The primer sequences will be made available upon request.

### Mitochondrial enzymatic activity

Inguinal white adipose tissue was disrupted in 0.1 M potassium phosphate buffer with 5 mM EDTA using a Dounce homogenizer. The homogenate was centrifuged at 10,000 x g for 10 min at 4°C to remove the fat, and the supernatant was used for protein quantification (BCA kit, Pierce) and determination of citrate synthase and electron transport activities. Citrate synthase was determined as previously described [48] using 3-10 μg of total homogenate. Activity of complexes II-III or I-III was assayed as described in [49], using 80-150 μg of total homogenate and succinate or NADH as substrates, respectively. The rate of cytochrome c reduction that was insensitive to antimycin A was subtracted from all measurements.

### Substrate oxidation

Valine oxidation into CO_2_ or α-ketoisovaleric acid (αKIV) was measured according to [12] with slight modifications. Palmitate oxidation and incorporation into lipids was measured as described previously [50]. Briefly, tissues were minced, weighed and placed into glass tubes (approximately 10 mg per tube) containing 0.5 ml of Krebs-Ringer bicarbonate buffer [in mM: 118 NaCl, 4.8 KCl, 1.25 CaCl_2_, 1.2 KH_2_PO_4_, 1.2 MgSO_4_, 25 NaHCO_3_, 10 HEPES (pH 7.4)]. For valine assays, the buffer was supplemented with 5 mM glucose and 1 mM valine containing 0.2 μCi [U-^14^C] valine per tube. For palmitate assays, the buffer was supplemented with 100 μM palmitate containing 0.2 μCi [U-^14^C] palmitate per tube. For adipose tissue samples, the buffer was also supplemented with 2% (w/v) BSA (fatty acid free). For muscle samples, the buffer was supplemented with 0.2% (w/v) BSA. A dry piece of Whatman paper was placed inside the tube around its upper part, with no direct contact with the buffer, in order to absorb the CO_2_ produced. The tubes were sealed with parafilm and tissue explants were incubated with shaking at 37°C. After 1h, the reaction was terminated with the injection of 200 μL of 6N sulfuric acid into the reaction mixture and 200 μL of phenyletilamine:methanol (1:1) onto the Whatman paper. After 20 min the paper was collected. In the valine assays, the paper was replaced and the tubes were resealed. Hydrogen peroxide (200 μL, 30% w/v) was injected into the reaction mixture and 200 μL of phenyletilamine:methanol (1:1) onto the paper for collecting the CO_2_ generated by the decarboxylation of αKIV. Explants were removed and destined to lipid extraction with 1 mL of chloroform:methanol (2:1). ^14^C radioactivity was assessed in the papers or lipid extracts and normalized by tissue weight.

### Oxygen consumption

Oxygen consumption was performed in isolated adipocytes using the Oroboros O2k oxygraph. Adipocytes from the inguinal fat depots were isolated and respiratory rate was measured as previously described [51, 52]. Adipocytes from 3-5 mice per group were pooled in KRP buffer (130 mM NaCl, 4.7 mM KCl, 1.24 mM MgSO_4_, 2.5 mM CaCl_2_, 10 mM HEPES, 2.5 mM NaH_2_PO_4_, 5 mM D-glucose, 2% BSA). Respiration was measured in KRP devoid of BSA. 1 mM succinate and 0.5 μg/ml antimycin A were added sequentially. Respiration before addition of succinate was subtracted from all rates. Succinate-dependent respiration was obtained by subtracting antimycin A-insensitive respiratory rate. Values were normalized by the number of cells and expressed as percentage of Lox.

### Biochemical analyses

For lactate and ATP assays, tissues were homogenized in 4% trichloroacetic acid and subjected to two rounds of centrifugations for 5 min at 13,000 g and 4°C. The supernatant had the pH adjusted to 7.0 and was used for lactate quantitation as described in [53] or ATP measurement using an ATP Bioluminescent Assay Kit from Sigma-Aldrich. NAD^+^ and NADH were measured using the NAD/NADH Assay Kit from Bioassays. Values were normalized by tissue weight. For glutathione determination, tissue homogenates (diluted in 0.1 M potassium phosphate buffer/5 mM EDTA) were de-proteinized by addition of 0.6% sulfosalycilic acid/0.1% Triton X-100 and centrifugation for 10 min at 8,000 x g and 4°C. The supernatant was used for glutathione determination as described in [54]. Glutathione levels were normalized by the protein content in the pre de-proteinized aliquot, as measured by the BCA kit (Pierce).

### Protein carbonylation

One volume of protein extract (maximum protein concentration of 4 mg/mL) was mixed with one volume of 24% SDS and one volume of 40 mM 2,4-dinitrophenylhydrazine solution. The mixture was incubated for 30 min at room temperature in the dark. A neutralizing solution containing 2 M Tris, 30% Glycerol and 19% mercaptoethanol was titrated into the mixture until it became orange. The neutralized mixture was then subjected to western blotting using equal amounts of protein and an antibody targeting dinitrophenyl (D9656) from Sigma-Aldrich.

### Microscopy

Microscopy analyses were performed as previously described [55]. Briefly, mice were anesthetized and transcardiacally perfused for 5-8 min with 2% glutaraldehyde in 0.1 M phosphate buffer (pH 7.4). After perfusion, WAT was isolated and kept in 0.1% glutaraldehyde/0.1 M phosphate buffer (pH 7.4). Thin sections were obtained with an MT-X ultratome (RMC). Slices were separated for histological analyses and stained with hematoxylin and eosin (H&E). For transmission electron microscopy, slices were stained with lead citrate and examined with a CM10 transmis-sion electron microscope (Philips). Morphometric evaluation of adipocytes was performed blindly using image analysis software (Lucia IMAGE Software, Laboratory Imaging). H&E images were used for determination of mean adipocyte size and adipocyte type.

### Insulin sensitivity and glucose tolerance

Glucose and insulin tolerance tests were performed one week prior to the euthanasia and followed the protocols described elsewhere [16]. Briefly, mice were injected intraperitoneally with glucose (1 g/kg body weight) after overnight fasting or insulin (0.75U/kg body weight; Humalog, Eli Lilly) after 2h fasting. Blood samples were collected at the indicated time-points through a small cut at the tail tip of the mouse and glucose levels were measured using a glucometer (Accu-Chek, Roche). When indicated, rapamycin (LC laboratories) was given i.p. (5 mg/kg body weight in 0.2% methylcellulose) 2h prior to the insulin injection. To assess insulin signaling, mice were fasted overnight, anesthetized and injected with a bolus of insulin (10 U) into the inferior vena cava. After 5 min, mice were euthanized and WAT was collected. Phosphorylation of Akt and Erk was determined by western blotting.

### Life expectancy

Kaplan-Meier curves were determined for *ad libitum*, chow diet fed male and female mice. Median lifespan was assessed using GraphPrism and Gompertz modeling using JMP.

### Statistical analysis

Results are expressed as the mean ± standard error of the mean (SEM) unless indicated otherwise. We used Student t test to compare two independent groups and ANOVA to compare more than two groups. Two-way ANOVA was used when data had more than one categorical independent variable. We used the MetaboAnalyst package of statistical tools to analyze and test the metabolomic data. Statistical significance was consider when P < 0.05.

## SUPPLEMENTARY DATA FIGURES AND TABLE


