# Supplementary material for: Fat-specific Dicer deficiency accelerates aging and mitigates several effects of dietary restriction in mice
Source: Aging (Albany NY). 2016 May 28;8(6):1201–15. doi: 10.18632/aging.100970 (PMC4931827; doi:10.18632/aging.100970)
Supplement: Supplementary file 1 [file aging-08-1201-s001.pdf]

SUPPLEMENTARY DATA

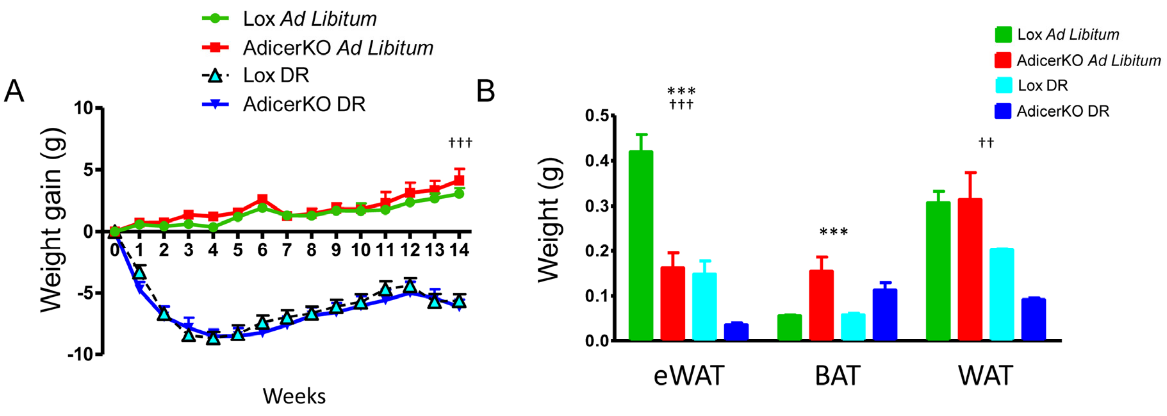

Supplementary Figure 1. Body weight and fat mass of AdicerKO mice. Twelve-week old mice were subjected to *ad libitum* (AL) or dietary restriction (DR) regimens for three months. (**A**) Body weight. (**B**) Fat mass (N=4-8 per condition). Mean ± SEM. *** P < 0.001 for genotype effect. †† P < 0.01, ††† P < 0.001 for diet effect. WAT, inguinal white adipose tissue. eWAT, epididymal white adipose tissue. BAT, interscapular brown adipose tissue.

Supplementary Table 1. Pathway analysis of serum metabolomics data comparing all conditions. Differentially expressed pathways when all conditions were compared (Lox AL, AdicerKO AL, Lox DR and AdicerKO DR).

Pathway Name	*Total*	*Hits*	*p*	*FDR*
Fatty acid metabolism	***39***	***1***	***0.012752***	***0.12663***
Valine, leucine and isoleucine biosynthesis	***11***	***3***	***0.017801***	***0.12663***
Valine, leucine and isoleucine degradation	***38***	***3***	***0.017801***	***0.12663***
Pantothenate and CoA biosynthesis	***15***	***1***	***0.025044***	***0.12663***
Glycerophospholipid metabolism	***30***	***2***	***0.026662***	***0.12663***
Phenylalanine, tyrosine and tryptophan biosynthesis	***4***	***2***	***0.033377***	***0.12663***
Phenylalanine metabolism	***11***	***2***	***0.033377***	***0.12663***
Aminoacyl-tRNA biosynthesis	***69***	***17***	***0.04653***	***0.12663***
Selenoamino acid metabolism	***15***	***1***	***0.047154***	***0.12663***
Taurine and hypotaurine metabolism	*8*	*1*	*0.053879*	*0.12663*
Primary bile acid biosynthesis	*46*	*1*	*0.053879*	*0.12663*
Tyrosine metabolism	*44*	*1*	*0.056882*	*0.12663*
Ubiquinone and other terpenoid-quinone biosynthesis	*3*	*1*	*0.056882*	*0.12663*
Arachidonic acid metabolism	*36*	*1*	*0.058074*	*0.12663*
Linoleic acid metabolism	*6*	*1*	*0.058074*	*0.12663*
alpha-Linolenic acid metabolism	*9*	*1*	*0.058074*	*0.12663*
Histidine metabolism	*15*	*4*	*0.059795*	*0.12663*

Arginine and proline metabolism	*44*	*9*	*0.18956*	*0.37912*
Cysteine and methionine metabolism	*27*	*2*	*0.21403*	*0.40408*
Glycine, serine and threonine metabolism	*31*	*2*	*0.22678*	*0.40408*
Alanine, aspartate and glutamate metabolism	*24*	*5*	*0.2624*	*0.40408*
Lysine biosynthesis	*4*	*1*	*0.27126*	*0.40408*
Biotin metabolism	*5*	*1*	*0.27126*	*0.40408*
Tryptophan metabolism	*40*	*3*	*0.27569*	*0.40408*
Methane metabolism	*9*	*1*	*0.30306*	*0.40408*
Cyanoamino acid metabolism	*6*	*1*	*0.30306*	*0.40408*
Sphingolipid metabolism	*21*	*1*	*0.30306*	*0.40408*
beta-Alanine metabolism	*17*	*3*	*0.37459*	*0.47022*
Lysine degradation	*23*	*2*	*0.37879*	*0.47022*
Glutathione metabolism	*26*	*4*	*0.39554*	*0.47464*
Nitrogen metabolism	*9*	*3*	*0.5897*	*0.68481*
Butanoate metabolism	*22*	*1*	*0.63277*	*0.6903*
Porphyrin and chlorophyll metabolism	*27*	*1*	*0.63277*	*0.6903*
D-Glutamine and D-glutamate metabolism	*5*	*2*	*0.84868*	*0.89236*
Purine metabolism	*68*	*1*	*0.89236*	*0.89236*
Pyrimidine metabolism	*41*	*1*	*0.89236*	*0.89236*

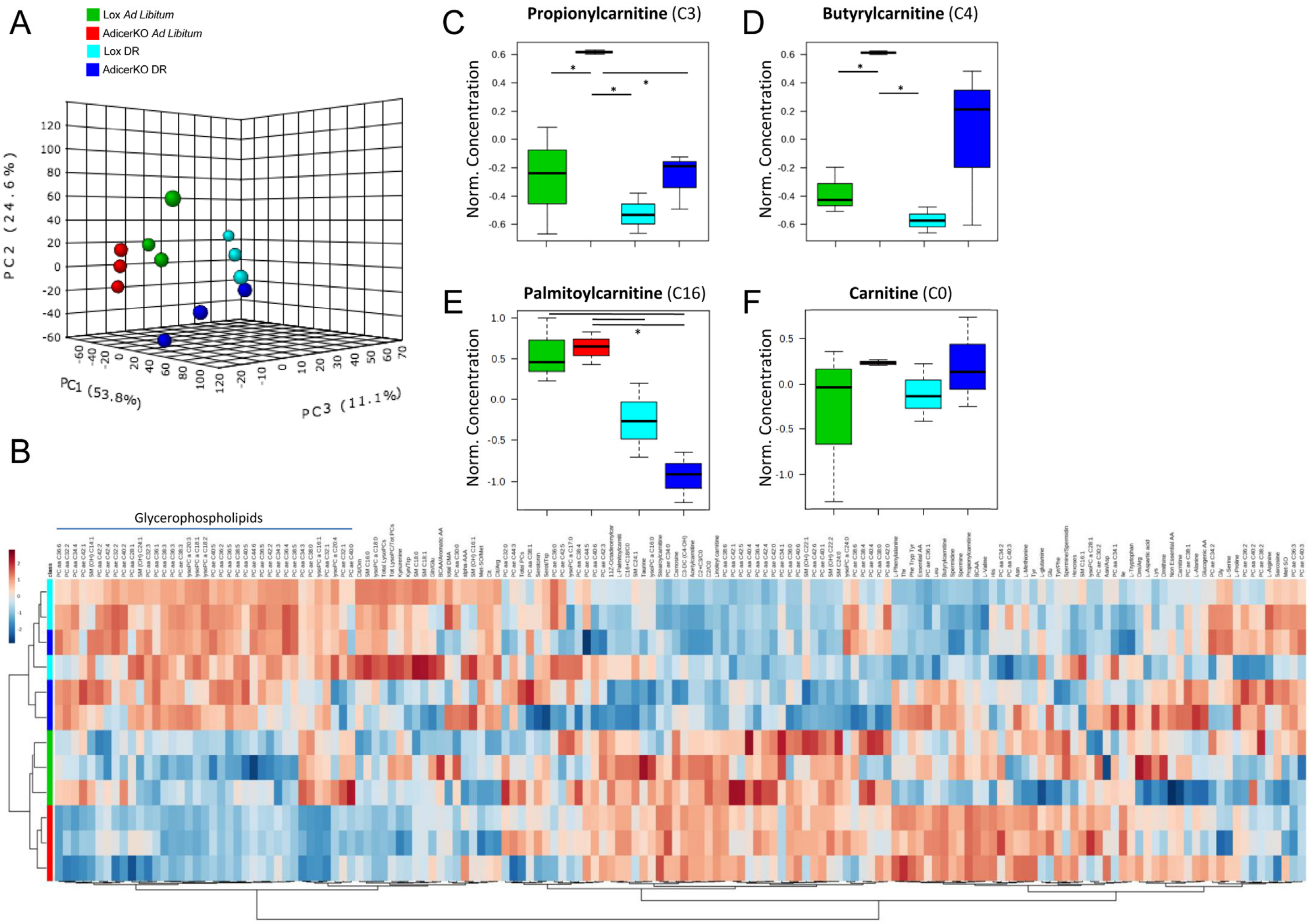

Supplementary Figure 2. Serum metabolite changes in AdicerKO mice. Twelve-week old mice were subjected to *ad libitum* (AL) or dietary restriction (DR) regimens for three months. Mice were euthanized at the end of the protocol after overnight fasting and metabolomics was conducted in the serum samples. Values were normalized by the average of the Lox AL group, Log2 transformed and Pareto scaled. Data was subjected to (**A**) Partial Least Squares Discriminant Analysis (PLS-DA) and (**B**) Hierarchical Clustering Analysis. (C-F) Selected metabolites. N=3 per condition. Data are mean ± SE. * $P < 0.05$.

Supplementary Table 2. Pathway analysis of serum metabolomics data in AdicerKO mice. Differentially expressed pathways when Lox *ad libitum* and AdicerKO *ad libitum* were compared.

Pathway Name	*Total*	*Hits*	*p*	*FDR*
Valine, leucine and isoleucine biosynthesis	***11***	***3***	***2.20E-04***	***0.0039647***
Valine, leucine and isoleucine degradation	***38***	***3***	***2.20E-04***	***0.0039647***
Phenylalanine, tyrosine and tryptophan biosynthesis	*4*	*2*	*0.058927*	*0.44812*
Phenylalanine metabolism	*11*	*2*	*0.058927*	*0.44812*
Selenoamino acid metabolism	*15*	*1*	*0.078201*	*0.44812*
Aminoacyl-tRNA biosynthesis	*69*	*17*	*0.0869*	*0.44812*
Tyrosine metabolism	*44*	*1*	*0.099581*	*0.44812*
Ubiquinone and other terpenoid-quinone biosynthesis	*3*	*1*	*0.099581*	*0.44812*
Pantothenate and CoA biosynthesis	*15*	*1*	*0.11684*	*0.46735*
Alanine, aspartate and glutamate metabolism	*24*	*5*	*0.21123*	*0.7081*
Tryptophan metabolism	*40*	*3*	*0.21636*	*0.7081*
Cysteine and methionine metabolism	*27*	*2*	*0.25371*	*0.73455*
Arachidonic acid metabolism	*36*	*1*	*0.30606*	*0.73455*
Linoleic acid metabolism	*6*	*1*	*0.30606*	*0.73455*
alpha-Linolenic acid metabolism	*9*	*1*	*0.30606*	*0.73455*
Glycerophospholipid metabolism	*30*	*2*	*0.37639*	*0.77163*
Histidine metabolism	*15*	*4*	*0.38953*	*0.77163*
Nitrogen metabolism	*9*	*3*	*0.41275*	*0.77163*
beta-Alanine metabolism	*17*	*3*	*0.41291*	*0.77163*
Arginine and proline metabolism	*44*	*9*	*0.48213*	*0.77163*
Lysine degradation	*23*	*2*	*0.49067*	*0.77163*
Glutathione metabolism	*26*	*4*	*0.50355*	*0.77163*
Purine metabolism	*68*	*1*	*0.53907*	*0.77163*
Pyrimidine metabolism	*41*	*1*	*0.53907*	*0.77163*
Butanoate metabolism	*22*	*1*	*0.57659*	*0.77163*
Porphyrin and chlorophyll metabolism	*27*	*1*	*0.57659*	*0.77163*
D-Glutamine and D-glutamate metabolism	*5*	*2*	*0.57872*	*0.77163*
Methane metabolism	*9*	*1*	*0.66013*	*0.79215*
Cyanoamino acid metabolism	*6*	*1*	*0.66013*	*0.79215*
Sphingolipid metabolism	*21*	*1*	*0.66013*	*0.79215*
Glycine, serine and threonine metabolism	*31*	*2*	*0.69424*	*0.80621*
Fatty acid metabolism	*39*	*1*	*0.76257*	*0.85789*
Taurine and hypotaurine metabolism	*8*	*1*	*0.89892*	*0.92402*
Primary bile acid biosynthesis	*46*	*1*	*0.89892*	*0.92402*
Lysine biosynthesis	*4*	*1*	*0.92402*	*0.92402*
Biotin metabolism	*5*	*1*	*0.92402*	*0.92402*

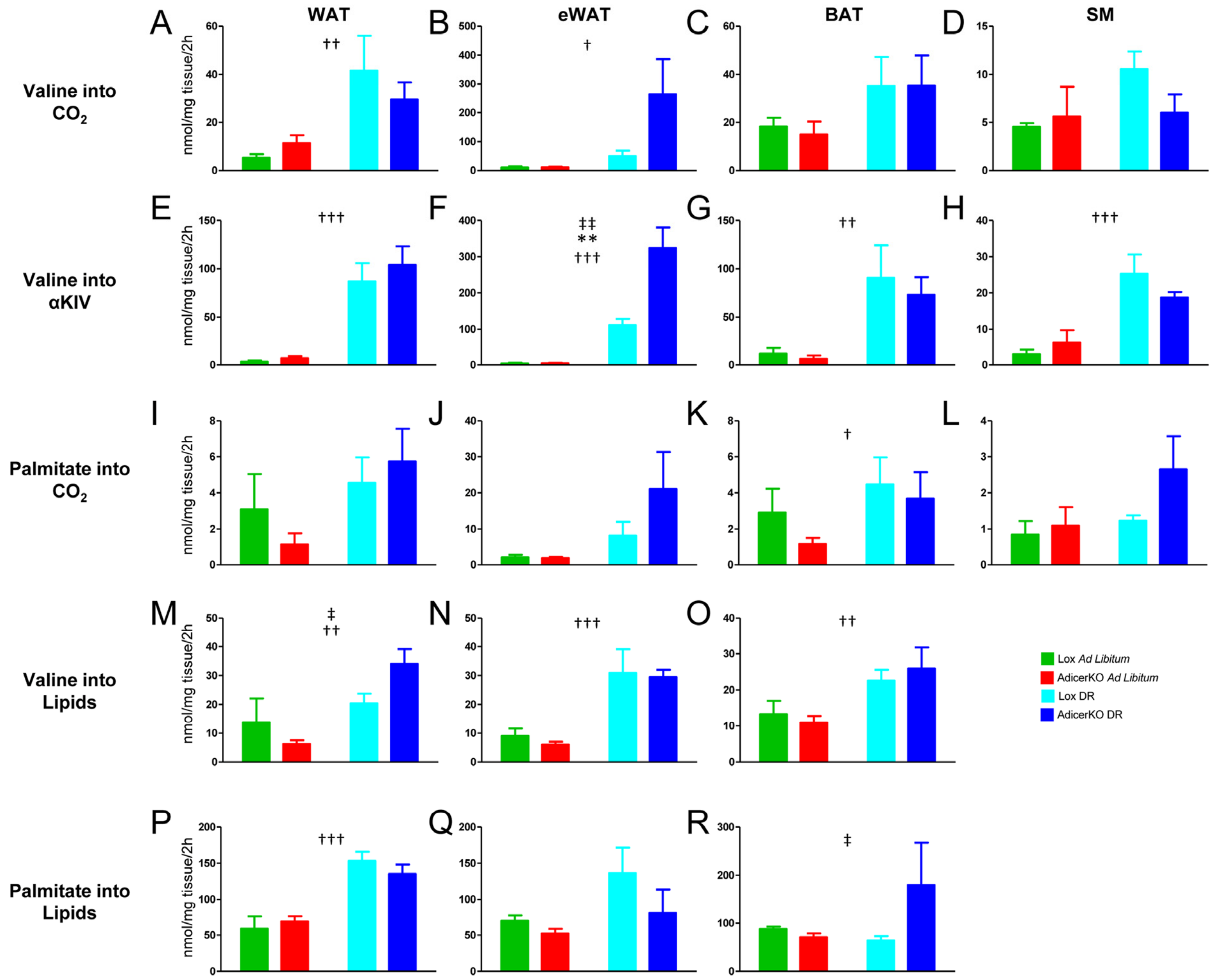

Supplementary Figure 3. Substrate oxidation and lipid synthesis in WAT of AdicerKO mice. Twelve-week old mice were subjected to ad libitum (AL) or dietary restriction (DR) regimens for one month. At the end of the protocol, tissues were isolated, minced and explants were used to assess (**A**-**D**) complete oxidation of valine, (**E**-**H**) oxidation of valine into α-ketoisovaleric acid (αKIV), (**I**-**L**) complete oxidation of palmitate, (**M**-**O**) lipid synthesis from valine, and (**P**-**R**) lipid synthesis from palmitate. WAT, inguinal white adipose tissue. eWAT, epididymal white adipose tissue. BAT, interscapular brown adipose tissue. SM, gastrocnemius skeletal muscle. N=3-5 per condition. Mean ± SEM. ** $P < 0.01$ for genotype effect; † $P < 0.05$, †† $P < 0.01$, ††† $P < 0.001$ for diet effect; ‡ $P < 0.05$, ‡‡ $P < 0.01$ for diet-genotype interaction.

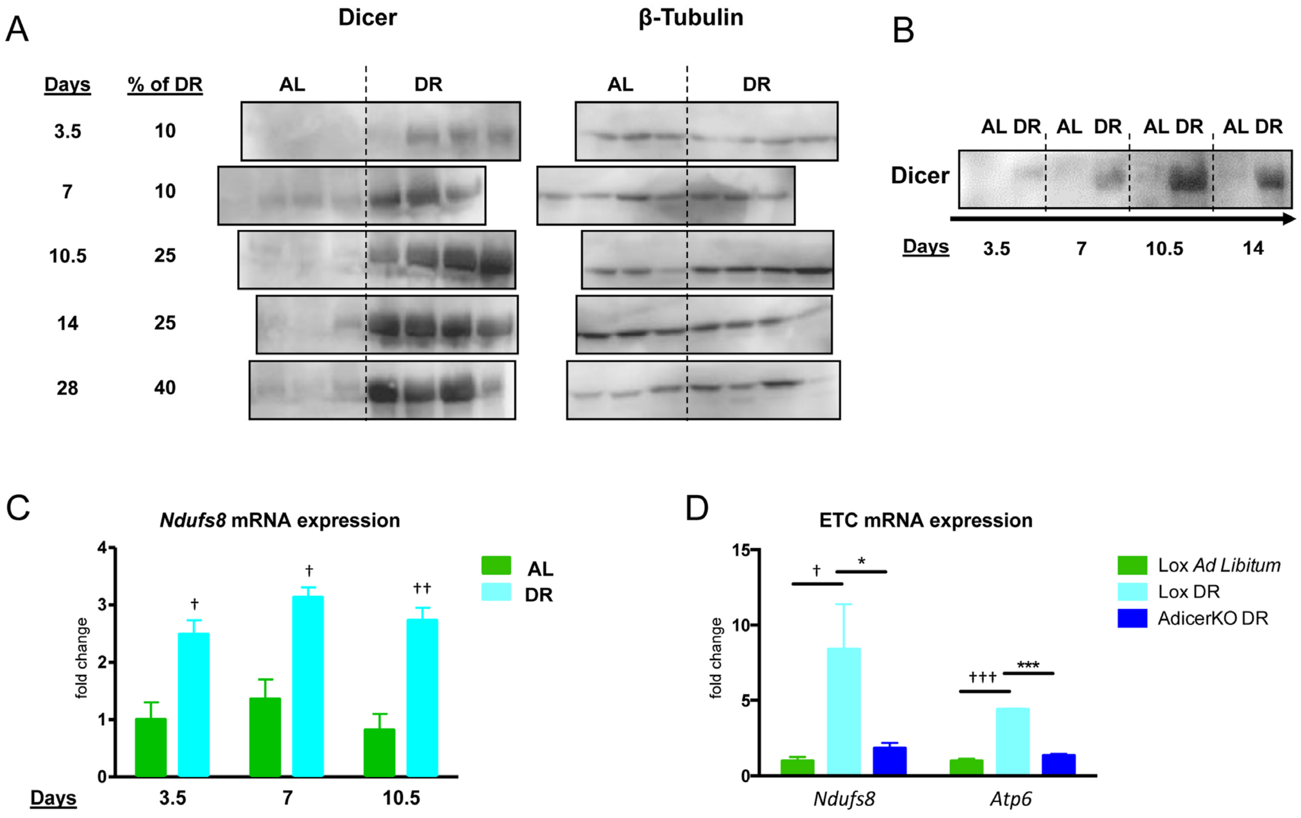

Supplementary Figure 4. Kinetics of Dicer and mitochondrial gene expression in WAT upon DR. Twelve-week old C57BL/6 mice were subjected to *ad libitum* (AL) or dietary restriction (DR) regimens for up to one month. Mice were euthanized at different time-points and WAT was collected. (**A**) Western blotting for Dicer and β-tubulin. (**B**) Representative blot. (**C,D**) Gene expression of components of the Electron Transport Chain (ETC) was assessed by RT-qPCR (N=3-5 per condition). Mean ± SEM. * $P < 0.05$, *** $P < 0.05$ for genotype effect; † $P < 0.05$, †† $P < 0.01$, ††† $P < 0.001$ for diet effect.

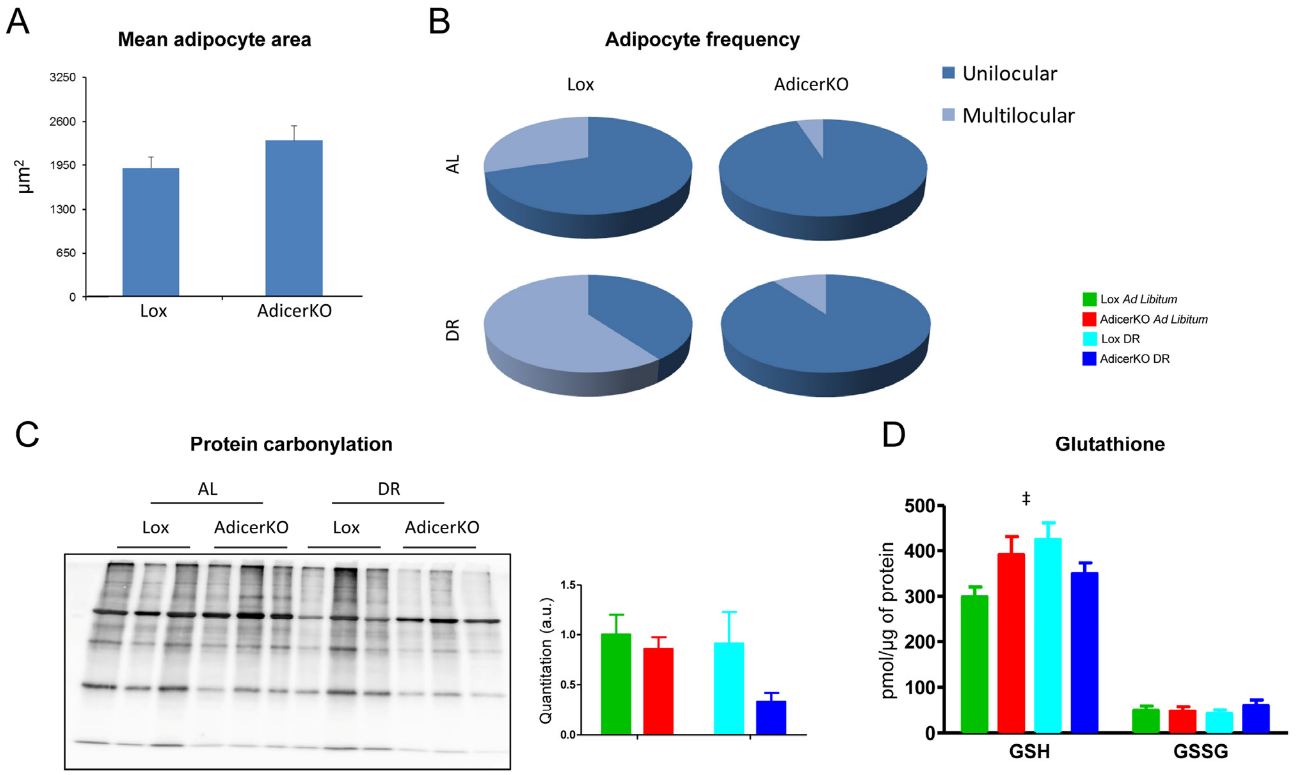

Supplementary Figure 5. Adipocyte size and redox balance of WAT of AdicerKO mice. Twelve-week old mice were subjected to *ad libitum* (AL) or dietary restriction (DR) regimens for one month and WAT was isolated. (**A,B**) Tissue was processed for H&E staining and (**A**) mean adipocyte size and (**B**) adipocyte type were blindly assessed using microscopic images (N=4-5 animals per condition, one image per animal collected always in the same anatomical location, *i.e.* around the inguinal lymph node). (**C**) Overall protein carbonylation was assessed by immunoblot and quantitated (right panel) using image densitometry. (**D**) Reduced (GSH) and oxidized (GSSG) glutathione levels were measured in tissue extracts (N=3-5 animals per condition). Mean ± SEM. ‡ P < 0.05 for diet-genotype interaction.

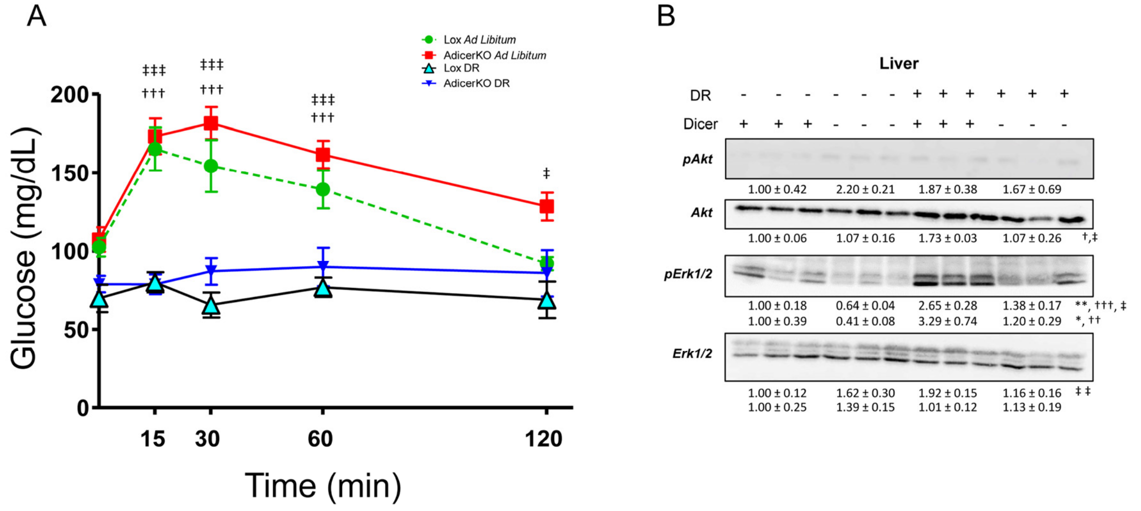

Supplementary Figure 6. Glucose tolerance test and liver insulin signaling in AdicerKO mice. Twelve-week old mice were subjected to *ad libitum* (AL) or dietary restriction (DR) regimens for three (**A**) or one (**B**) month. (**A**) Glucose tolerance test was performed one week prior to the end of the protocol (N=5-7 per condition). Mean ± SEM. ††† P < 0.001 Lox AL vs. Lox DR; ‡ P < 0.05, ‡‡‡ P < 0.001 AdicerKO AL vs. AdicerKO DR. (**B**) Western blots of liver extracts pAkt, phospho-Akt. pErk1/2, phospho-Erk1/2. Numbers are quantitation of blots (fold expression in comparison to control group) ± SEM. * P < 0.05, ** P < 0.01 for genotype effect; †† P < 0.01, ††† P < 0.001 for diet effect; ‡ P < 0.05, ‡‡ P < 0.01 for diet-genotype interaction.
